# New Generation of Orthodontic Elastomeric Ligature to Prevent Enamel Demineralization In Vivo

**DOI:** 10.3390/ijms25158409

**Published:** 2024-08-01

**Authors:** Ce Bian, Menghao Lyu, Mengyao Zhu, Chaoran Yu, Yiman Guo, Michael D. Weir, Radi Masri, Yuxing Bai, Hockin H. K. Xu, Ning Zhang

**Affiliations:** 1 Department of Orthodontics, School of Stomatology, Capital Medical University, Beijing 100050, China; 2 Department of Periodontics, School of Stomatology, Capital Medical University, Beijing 100050, China; 3 Department of Biomaterials and Regenerative Dental Medicine, University of Maryland School of Dentistry, University of Maryland, Baltimore, MD 21201, USA; 4 Department of Advanced Oral Sciences and Therapeutics, University of Maryland School of Dentistry, University of Maryland, Baltimore, MD 21201, USA

**Keywords:** enamel demineralization, orthodontics, elastomeric ligature, antibacterial properties, mechanical properties

## Abstract

This study aimed to synthesize a novel elastomeric ligature with dimethylaminohexadecyl methacrylate (DMAHDM) grafted, providing a new strategy for improving the issue of enamel demineralization during fixed orthodontics. DMAHDM was incorporated into elastomeric ligatures at different mass fractions using ultraviolet photochemical grafting. The antibacterial properties were evaluated and the optimal DMAHDM amount was determined based on cytotoxicity assays. Moreover, tests were conducted to evaluate the in vivo changes in the mechanical properties of the elastomeric ligatures. To assess the actual in vivo effectiveness in preventing enamel demineralization, a rat demineralization model was established, with analyses focusing on changes in surface microstructure, elemental composition, and nanomechanical properties. Elastomeric ligatures with 2% DMAHDM showed excellent biocompatibility and the best antibacterial properties, reducing lactic acid production by 65.3% and biofilm bacteria by 50.0% within 24 h, without significant mechanical property differences from the control group (*p* > 0.05). Most importantly, they effectively prevented enamel demineralization in vivo, enhancing elastic modulus by 73.2% and hardness by 204.8%. Elastomeric ligatures incorporating DMAHDM have shown great potential for application in preventing enamel demineralization, providing a new strategy to solve this issue during fixed orthodontics.

## 1. Introduction

The most common side-effect of fixed orthodontic treatment is irreversible, unhealthy, and aesthetically displeasing enamel demineralization, known as white spot lesions (WSLs) [1]. WSLs can appear around brackets as early as four weeks after orthodontic treatment begins, with a reported prevalence rate of 75.6% among orthodontic patients [2]. Current measures to prevent enamel demineralization during orthodontic treatment have certain limitations in terms of effectiveness [3,4,5]. Thus, there is an urgent need to explore and investigate additional interventions and preventive strategies to address this widespread issue.

The initial factor in enamel demineralization is the formation and accumulation of plaque biofilm [6]. Acid-producing cariogenic bacteria within the plaque biofilm lower the pH of the tooth surface microenvironment, causing the enamel to lose calcium and phosphate ions, leading to demineralization. Therefore, using novel bioactive materials to inhibit plaque biofilm formation is an effective strategy for preventing enamel demineralization [2]. Studies have shown that dimethylaminohexadecyl methacrylate (DMAHDM), with a carbon chain length of sixteen, has the strongest antibacterial efficacy among quaternary ammonium salts (QAS) monomers [7]. In our previous study, we incorporated DMAHDM into orthodontic cement and observed a three-log reduction in plaque biofilm compared to the control group [8]. However, clinical observations often reveal enamel demineralization around brackets [9]. The limited interaction between orthodontic adhesives and the surrounding plaque constrains the optimal antibacterial effects of DMAHDM due to its contact-killing mechanism [10].

Elastomeric ligatures, as an essential tool in orthodontic treatment, serve to tightly secure the archwires to the bracket [2]. Polyurethane, as the main component, offers good processability, various processing methods, excellent toughness, and elasticity. However, the mechanical properties of the material can experience force degradation due to the oral environment and over time. Rapid force degradation can negatively impact its mechanical properties, leading to not fit between the archwires and bracket, ultimately affecting the efficacy of orthodontics. Therefore, the mechanical properties of elastomeric ligatures are particularly critical for successful orthodontic treatment [2].

Offering elastomeric ligatures with antimicrobial properties provides several advantages: They provide antimicrobial functionality without relying on patient compliance; they ensure precise antimicrobial effects by securing elastomeric ligatures around orthodontic brackets (which have a large surface area and directly contact the plaque); they significantly enhance antimicrobial efficacy and allow for replacement at each follow-up visit to achieve long-term, continuous, antimicrobial effects. Additionally, they help delay the degradation of mechanical properties, ensuring the effectiveness of orthodontic treatment. Despite efforts to incorporate fluoride or silver ions into elastomeric ligatures for antimicrobial effects, these effects are often transient, lasting only about a week due to limitations in coating methods and antimicrobial agents [11]. Therefore, exploring innovative methods that combine new bioactive materials with elastomeric ligatures is essential to address this issue [12].

Moreover, meeting the biocompatible standards is paramount for developing a novel bioactive material with practical clinical application value [13]. Biocompatibility analysis of new materials and medical devices is essential prior to their use in humans. Currently, the majority of prior studies on the biocompatibility of new materials have been conducted in vitro [14]; however, in vitro studies alone are not sufficient to fully understand the biocompatibility of these materials in complex living organisms. Animal models offer a more comprehensive alternative, allowing researchers to evaluate the biocompatibility and safety of novel materials in a living system that closely mimics human physiology [15]. Among various animal models, rat models are particularly favored by researchers due to ethical and cost considerations. Rats are more readily available, easier to handle, and less expensive to maintain compared to larger animals such as monkeys, dogs, and ferrets [16]. Additionally, the use of rat models raises fewer ethical concerns, making them a more practical and humane choice for preliminary biocompatibility testing [17].

While the formation and accumulation of dental plaque biofilm are the initiating factors for enamel demineralization, the complexity of the oral environment and the resilience of dental plaque biofilm pose significant challenges [18]. The practical antimicrobial activity of new materials can be influenced by the actual oral environment, leading to cases where enamel demineralization still occurs despite the use of these materials [19]. The current research lacks comprehensive studies on the practical efficacy of new bioactive materials in preventing enamel demineralization, with even fewer studies conducted in vivo [20]. This gap highlights the need for more thorough investigations into how these materials perform in real oral conditions. Rat molars, which share anatomical, biological, and physiological similarities with human molars are considered suitable models for conducting such studies [21]. Using rat molars can provide valuable insights into the practical effectiveness of bioactive materials in preventing enamel demineralization within the complex environment of the oral cavity.

This study employed ultraviolet photochemical grafting to incorporate DMAHDM into the elastomeric ligature to form a novel one, followed by the establishment of a rat enamel demineralization model to evaluate the actual in vivo preventive effect of the novel elastomeric ligature on enamel demineralization. The following hypotheses were proposed: (1) compared to the control group, the incorporation of DMAHDM will significantly reduce the plaque biofilms without causing cytotoxicity of the elastomeric ligatures; (2) the addition of DMAHDM will not negatively impact the mechanical properties and biocompatibility of the elastomeric ligatures; (3) the elastomeric ligatures containing DMAHDM will effectively prevent enamel demineralization in vivo.

## 2. Results

### 2.1. Analysis of the First Hypothesis

#### 2.1.1. Incorporation of DMAHDM with Elastomeric Ligatures

**Figure 1:**
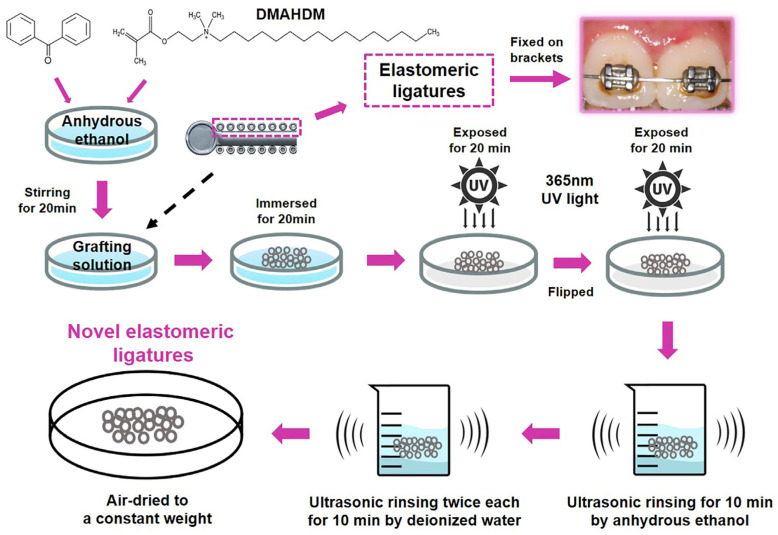
Ultraviolet (UV) photochemical grafting was employed to synthesize the novel elastomeric ligature.

**Figure 2:**
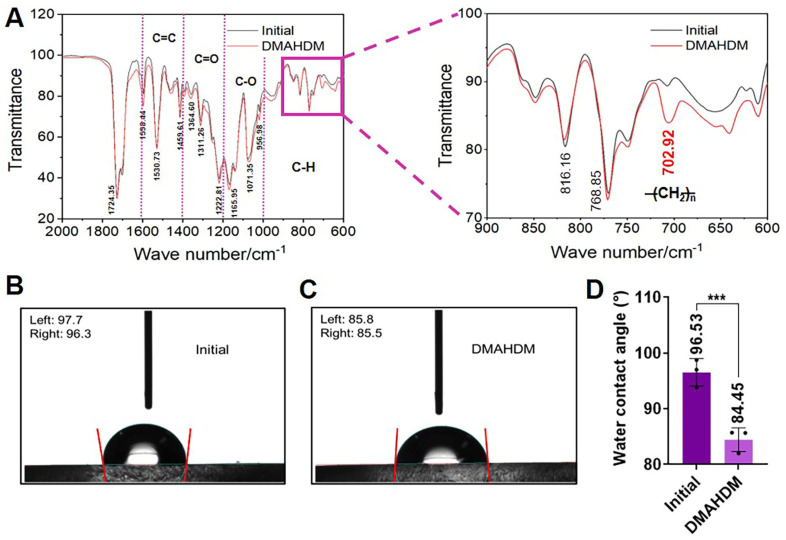
Characterization of the novel orthodontic elastomeric ligatures. (**A**) Fourier-transform infrared spectroscopy (FTIR) of the novel elastomeric ligature with the characteristic peak around 702 cm^−1^. (**B**,**C**) Water contact angle measurements of the initial and novel elastomeric ligatures. (**D**) The difference in water contact angle measurements between the initial and novel elastomeric ligatures. The data are represented as Mean ± SD (*n* ≥ 3). *** denoted significant statistical differences with the initial group (*p* < 0.001). With DMAHDM grafted, the elastomeric ligature exhibited a transition from hydrophobicity to heightened hydrophilicity.

The novel elastomeric ligatures were synthesized by ultraviolet (UV) photochemical grafting, as shown in Figure 1. The Fourier-transform infrared spectroscopy (FTIR) results shown in Figure 2A indicate a significant peak increase at 702 cm^−1^ after grafting DMAHDM, attributed to the elongated carbon chain structure (CH_2_)_n_ in DMAHDM. Additionally, water contact angle experiments demonstrated an increase in surface hydrophilicity for the novel elastomeric ligatures relative to the control group in Figure 2B–D (*p* < 0.05), providing further evidence of the successful grafting of DMAHDM.

#### 2.1.2. Antibacterial Properties

**Figure 3:**
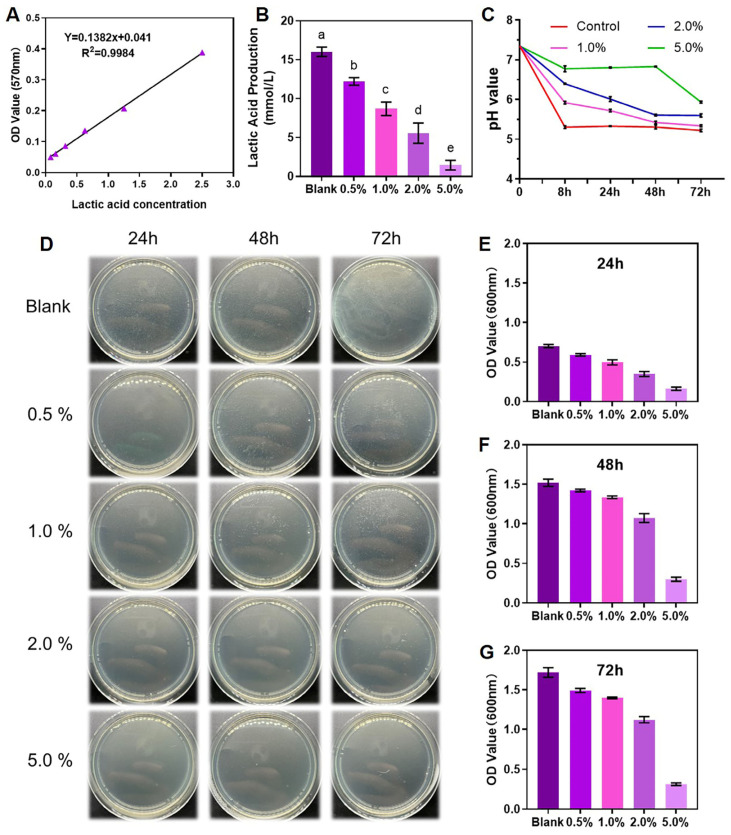
Antibacterial properties of the novel elastomeric ligatures. (**A**) The standard curve of lactic acid production. (**B**) The lactic acid production of *Streptococcus mutans* biofilms in 24 h. (**C**) Changes in the pH of the microenvironment after 8 h, 24 h, 48 h, and 72 h of culturing *Streptococcus mutans*. (**D**) Colony-forming unit counts of biofilms at 24, 48, and 72 h. (**E**–**G**) Spectrophotometer counting of biofilms at 24, 48, and 72 h. The 5% DMAHDM group exhibited optimal antibacterial efficacy, significantly suppressing the lactic acid production and the proliferation of biofilm microorganisms (*p* < 0.05). The data are represented as Mean ± SD (*n* ≥ 3). The different letters indicated significant differences (*p* < 0.05).

Table 1 and Figure 3 illustrate the antibacterial properties of the novel elastomeric ligatures. Utilizing the standard curve correlating lactate production with OD values (Figure 3A), the cumulative lactate production of the biofilms in each group over 24 h was calculated. As depicted in Figure 3B, an incremental addition of DMAHDM resulted in a gradual decline in biofilm lactate production, with the 5% DMAHDM group displaying the least lactate production (1.45 ± 0.63 mmol/L). Figure 3C showcases the fluctuations in pH of the culture medium post-cultivation for 8, 24, 48, and 72 h. It is evident that the 5% DMAHDM group exhibited optimal antibacterial efficacy, effectively mitigating changes in environmental pH and maintaining microenvironmental stability.

Furthermore, Figure 3D presents the colony-forming unit counts of biofilms at 24, 48, and 72 h, also indicating that the 5% DMAHDM group displayed the most robust antibacterial performance, significantly suppressing the proliferation of biofilm microorganisms. Figure 3E–G demonstrate the OD600 values measured using a spectrophotometer, revealing that, at equivalent time points, the 5% DMAHDM group exhibited the most potent antibacterial effect, with an 82% reduction compared to the control group at 72 h. Therefore, without considering biosafety concerns, the incorporation of 5% DMAHDM into the elastomeric ligatures conferred the highest level of antibacterial efficacy, with the 2% group ranking next in line.

#### 2.1.3. Cytotoxicity

**Figure 4:**
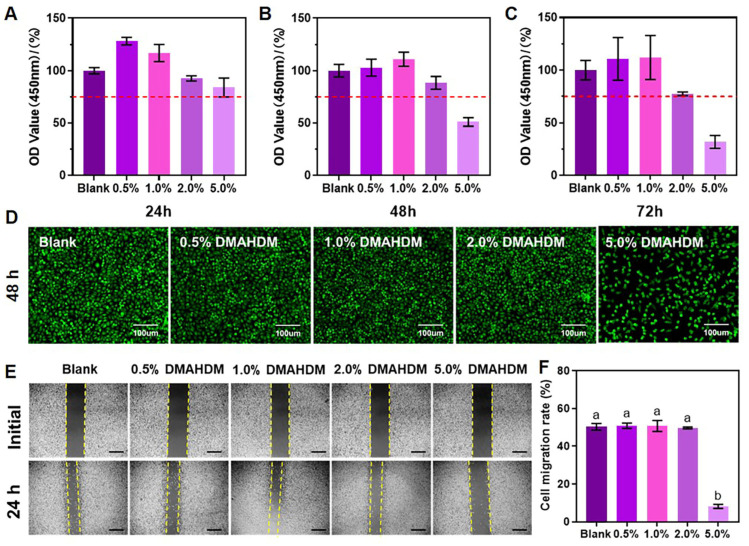
Cytotoxicity of the novel elastomeric ligatures. (**A**–**C**) Cell proliferation activity of L929 fibroblasts at 24, 48, and 72 h. (**D**) Fluorescence staining images of L929 viable cells cultured in the extracts from each group for 48 h (Scale bar = 100 μm). (**E**) Cell migration of L929 fibroblasts in each group. (**F**) Cell migration rate of L929 fibroblasts in each group. The elastomeric ligatures with 5% DMAHDM exhibited significant cytotoxicity compared to the other groups (*p* < 0.05), whereas those with 2% DMAHDM showed no cytotoxicity. The data were represented by Mean ± SD (*n* ≥ 3); different letters indicate significant differences (*p* < 0.05).

Table 2 and Figure 4A–C depict the effects of various concentrations of elastomeric ligature extracts on the proliferation of L929 fibroblast cells. It was observed that the elastomeric ligatures with 5% DMAHDM exhibited a significant reduction in cell proliferation activity over time, failing to meet the cytotoxicity requirements (75% of the blank control group). Conversely, the addition of 2% DMAHDM was found to be the highest concentration compliant with cytotoxicity standards.

Fluorescence staining images in Figure 4D similarly reveal a noticeable decrease in cell proliferation and abnormal cell morphology characterized by a rounded shape in the 5% DMAHDM group after 48 h of culture. In Figure 4E,F, it was observed that cell migration in the 5% DMAHDM extract group significantly slowed down compared to the other groups, where no noticeable effects were seen. Therefore, based on these findings, it could be concluded that elastomeric ligatures with 2% DMAHDM ensured both excellent cytotoxicity and the strongest antimicrobial performance. Ultimately, we determined that incorporating 2% DMAHDM was the optimal dosage for the novel orthodontic elastomeric ligatures.

### 2.2. Analysis of the Second Hypothesis

#### 2.2.1. Mechanical Properties of Elastomeric Ligatures

**Figure 5:**
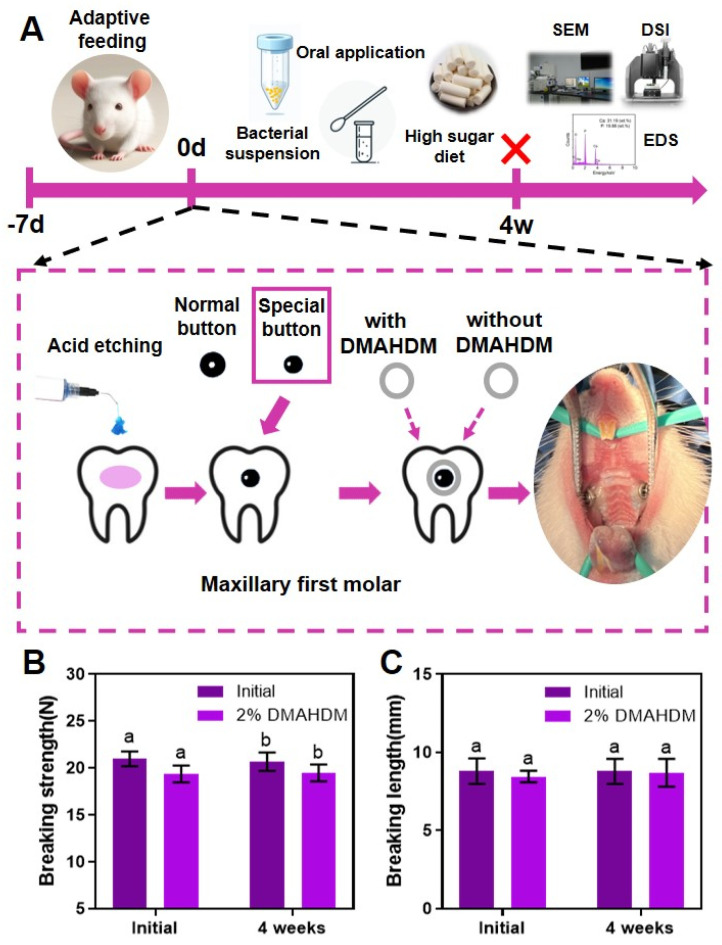
Establishment of the rat orthodontic enamel demineralization model and in vivo changes in the mechanical properties of the novel elastomeric ligatures: (**A**) establishment of the rat orthodontic enamel demineralization model; (**B**) breaking force of elastomeric ligatures in each group, initially and after 4 weeks of oral environment treatment; (**C**) breaking length of elastomeric ligatures in each group, initially and after 4 weeks of oral environment treatment. The addition of DMAHDM did not affect the mechanical properties of the elastomeric ligatures (*p* > 0.05). The data are represented as Mean ± SD (*n* = 6); different letters indicate significant differences (*p* < 0.05).

In Figure 5A, successful establishment of the rat orthodontic enamel demineralization model was illustrated. Changes in the breaking strength of the elastomeric ligatures before and after four weeks of in vivo treatment are depicted in Figure 5B, while Figure 5C shows the variation in the breaking length of the elastomeric ligatures before and after the same treatment period. The results indicated no significant differences in breaking force and breaking length between the control and experimental groups, both before and after the 4-week in vivo treatment, suggesting that the addition of DMAHDM did not compromise the original mechanical properties of the elastomeric ligatures.

#### 2.2.2. Mucosal Irritation

**Figure 6:**
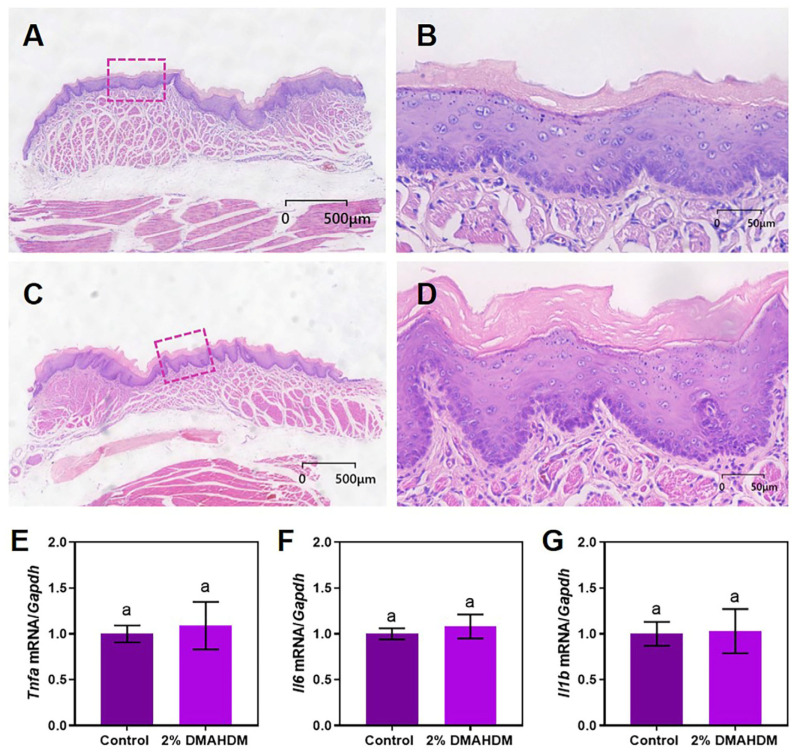
The biocompatibility of the novel elastomeric ligatures. (**A**) Hematoxylin and Eosin (HE)-stained image of oral mucosa from the control group rats (2000× magnification). (**B**) HE-stained image of the boxed area in (**A**) (20,000× magnification). (**C**) HE-stained image of oral mucosa from the DMAHDM group rats (2000× magnification). (**D**) HE-stained image of the boxed area in (**C**) (20,000× magnification). (**E**–**G**) Relative expression of mRNA of Tnfa, Il6, and Il1b in the oral mucosa of the control group and the DMAHDM group. The addition of 2% DMAHDM exhibited no effect on the biocompatibility of the elastomeric ligatures (*p* > 0.05). The data are represented as Mean ± SD (*n* ≥ 3); the same letter indicates no significant difference (*p* > 0.05).

Figure 6A and Figure 6C present the histological sections stained with HE from the oral mucosa tissues treated with extracts in the control and DMAHDM groups, respectively. Figure 6B and Figure 6D are separately magnified views of the boxed regions in Figure 6A and Figure 6C. The results revealed that the epithelium of the oral mucosa in both the control and DMAHDM groups remained continuously intact, with no infiltration of inflammatory cells beneath the epithelium, showing no significant differences between the two groups. Furthermore, analysis of PCR results in Figure 6E–G indicate no significant difference in the expression of tissue inflammatory cytokine markers Tnfa, Il6, and Il1b between them. This suggested that elastomeric ligatures containing 2% DMAHDM exerted no significant irritant effect on the oral mucosa, demonstrating good biocompatibility.

### 2.3. Analysis of the Third Hypothesis

#### 2.3.1. Surface Microscopy and Energy Spectrum Analysis

**Figure 7:**
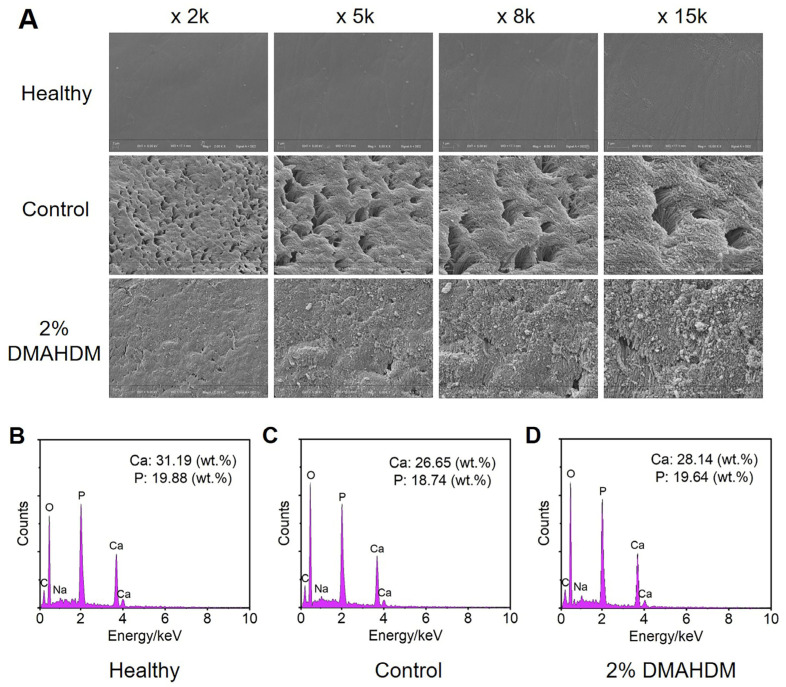
Effects of novel elastomeric ligatures on the surface morphology and elemental composition of rat molar enamel. (**A**) The SEM surface morphology of healthy enamel as well as enamels after one month of in vivo demineralization treatment in the control group and the 2% DMAHDM group. (**B**–**D**) The EDS elemental analysis of healthy enamel as well as enamels after one month of in vivo demineralization treatment in the control group and the 2% DMAHDM group. The incorporation of 2% DMAHDM into the elastomeric ligatures provided a preventive effect against enamel demineralization in vivo.

Figure 7 illustrates the surface morphology and elemental changes in the buccal enamel of the second molars in each group of rats. As shown in Figure 7A, the surface of healthy enamel was smooth and continuous, with orderly arranged enamel rods. After four weeks of enamel demineralization treatment, the control group exhibited numerous irregular pores on the enamel surface and high magnification revealed significant fragmentation and disordered arrangement of the enamel rods. In contrast, the enamel surface in the 2% DMAHDM group, while relatively rough, did not show extensive enamel rod breakage or irregular pores.

The elemental composition of healthy enamel, enamel treated with demineralization for 4 h in the control group, and enamel in the DMAHDM group are presented in Figure 7B–D. The results indicated that the mass ratios of calcium (Ca) and phosphorus (P) in both the control group and the 2% DMAHDM group were lower compared to healthy enamel, with the control group (Ca: 26.65 wt.%; P: 18.74 wt.%) showing a greater reduction than the 2% DMAHDM group (Ca: 28.14 wt.%; P: 19.64 wt.%). These demonstrated that adding 2% DMAHDM to elastomeric ligatures effectively reduced the rate of enamel demineralization, achieving a significant preventive effect against enamel demineralization.

#### 2.3.2. Nanoindentation Mechanical Properties

**Figure 8:**
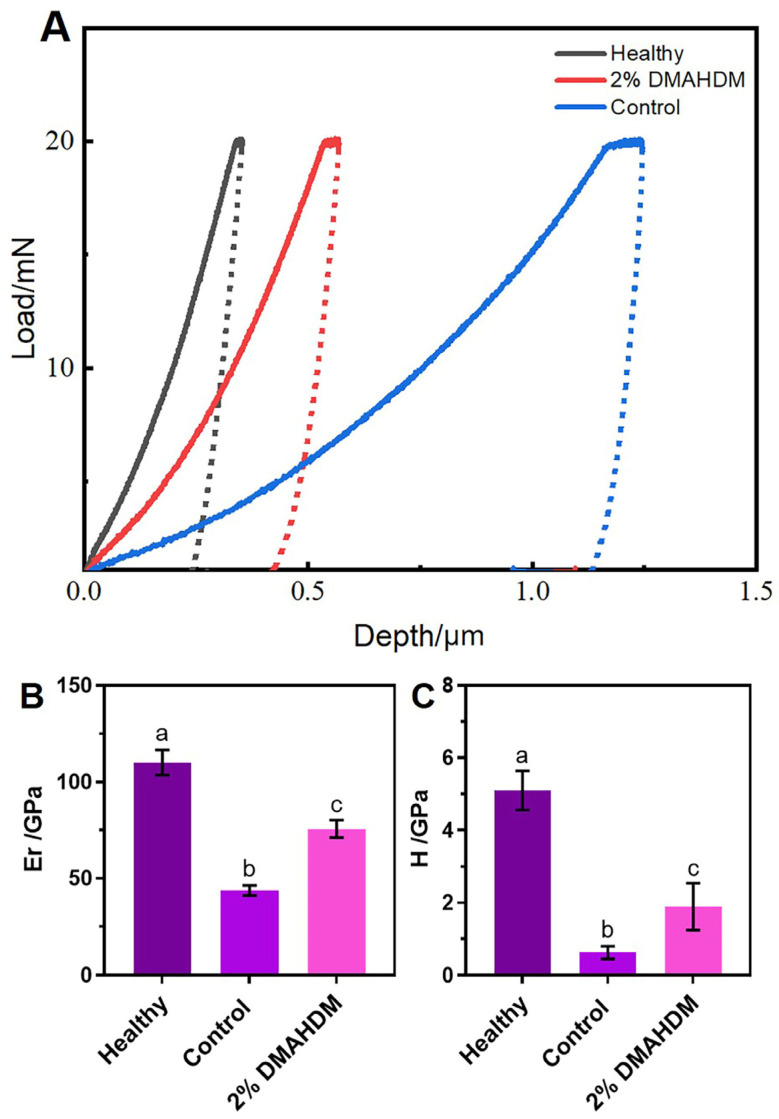
Effects of the novel elastomeric ligatures on mechanical properties of rat molar enamel. (**A**) The curve of load (mN) and indentation depth (μm) of different groups. With the same 25 mN load, the indentation depth of the novel elastomeric ligatures was less than that of the control ones. (**B**,**C**) The elastic modulus (Er) and hardness (H) of healthy enamel as well as enamels after one month of in vivo demineralization treatment in the control group and the 2% DMAHDM group. The data are represented as Mean ± SD (*n* = 6). Different letters indicate significant differences (*p* < 0.05). The elastomeric ligatures containing 2% DMAHDM showed a significant in vivo effect in preventing enamel demineralization.

The mechanical properties of the enamel in each group are presented in Table 3 and Figure 8. As shown in Figure 8A, under the same 25 mN load, after four weeks of demineralization treatment, the indentation depth of the enamel in both the control and DMAHDM groups was less than that of healthy enamel. However, the indentation depth of the enamel in the control group was significantly greater than that in the DMAHDM group. Figure 8B,C show that the elastic modulus (Er) and hardness (H) of the enamel in the control group were 43.65 ± 2.62 GPa and 0.62 ± 0.17 GPa, respectively, which were significantly lower than those in the DMAHDM group, 75.61 ± 4.57 GPa and 1.89 ± 0.65 GPa, respectively.

These results indicated that elastomeric ligatures containing 2% DMAHDM could improve the elastic modulus and hardness of enamel, slow down the deterioration of its mechanical properties, and obtain a significant preventive effect against enamel demineralization.

## 3. Discussion

As previously mentioned, giving orthodontic elastomeric ligatures antibacterial properties offers several advantages in preventing enamel demineralization compared to other antibacterial materials [22]. This study established a rat enamel demineralization model and, for the first time, investigated the impact of a novel orthodontic elastomeric ligature on inhibiting enamel demineralization in vivo. The results demonstrated that elastomeric ligatures containing 2% DMAHDM by mass significantly prevented the occurrence and progression of enamel demineralization. All three proposed hypotheses were confirmed: the addition of DMAHDM did not compromise the mechanical properties, cytotoxicity, or biocompatibility of the elastomeric ligatures; it reduced the formation of dental plaque biofilm in the oral cavity of rats; and the novel elastomeric ligatures containing DMAHDM significantly reduced enamel demineralization compared to the control group under in vivo conditions.

Quaternary ammonium salt (QAS) monomers, serving as organic antimicrobial agents, are renowned for their excellent biocompatibility and rapid, robust, broad-spectrum antibacterial effects, capable of eliminating hundreds of bacterial strains and microorganisms within minutes [23]. Their antibacterial mechanism involves the contact between the positively charged nitrogen atom (N^+^) and bacterial cell membranes, causing osmotic imbalance similar to puncturing a balloon with a needle, resulting in cell rupture and eventual death [24]. DMAHDM, a specific type of QAS monomer, exhibits potent antibacterial properties due to its extended carbon chain containing 16 carbons [25]. In this study, the novel elastomeric ligatures containing 2% DMAHDM reduced lactic acid production by 65.3% and decreased the total bacterial count by 50.0% within 24 h, demonstrating significant antibacterial performance.

However, recent studies have indicated that despite the potent bactericidal effects of bioactive materials, they may not be sufficient to completely eliminate dental plaque biofilm [26]. This is primarily due to the dynamic complexity of the oral environment and the intricate interactions among various bacteria within the biofilm. The oral cavity harbors a diverse range of bacterial species, forming a complex microbial ecosystem where bacteria within the biofilm can protect each other, increasing their resistance to antimicrobial agents [27]. As the concentration of bioactive materials decreases or disappears, residual dental plaque biofilm can swiftly regenerate, posing a continuous threat to healthy enamel. This regenerative ability makes it difficult for even the most powerful antimicrobial agents to completely eradicate all pathogenic bacteria, thereby maintaining the risk of enamel demineralization and cavities [28].

These microorganisms perform various functions in the oral environment, encompassing both beneficial and harmful effects. Typically, the microbial community system maintains a dynamic equilibrium where beneficial and harmful bacteria compete and interact [29]. For example, *Streptococcus mutans* and *Lactobacillus* metabolize carbohydrates to produce lactic acid, creating an acidic environment conducive to enamel demineralization. Conversely, bacteria such as *Streptococcus sanguinis* and non-pathogenic *Actinomyces* can compete with cariogenic bacteria for nutrients and produce antimicrobial agents, thereby inhibiting the proliferation of cariogenic bacteria [30]. Therefore, the key to effectively preventing enamel demineralization lies in modulating the composition of microbial communities within the dental plaque biofilm, promoting the growth of non-cariogenic bacteria while suppressing the expansion of cariogenic bacteria, thus achieving lasting and effective preventive outcomes.

In previous studies, researchers established an in vitro multispecies biofilm model comprising *Streptococcus mutans*, *Streptococcus sanguinis,* and *Streptococcus gordonii* [31]. The results confirmed that DMAHDM not only possesses potent antibacterial properties but also demonstrates exceptional microbial community regulation capabilities. It effectively reduced the proportion of *Streptococcus mutans* within the biofilm while increasing the ratios of *Streptococcus sanguinis* and *Streptococcus gordonii*. Consequently, DMAHDM shows significant advantages in mitigating enamel demineralization. However, no studies have yet investigated the actual in vivo effectiveness of DMAHDM-containing elastomeric ligatures in preventing enamel demineralization.

The enamel morphology of rat molars has some similarities to human enamel, making it a useful model for studying dental biology and disease mechanisms [32]. Therefore, this study used rats as a research model, bonding lingual buttons to the buccal side of their maxillary first molars to secure the elastomeric ligatures. An enamel demineralization model was established by feeding the rats a bacterial suspension and applying *Streptococcus mutans*, effectively simulating the real environment of elastomeric ligatures used in humans. The results preliminarily validate the significant preventive effect of orthodontic elastomeric ligatures containing 2% DMAHDM against enamel demineralization, with elastic modulus and hardness raised by 73.2% and 204.8%, respectively. This has laid the groundwork for further exploration of the potential applications of this novel material in clinical orthodontic treatment, demonstrating its significant preventive benefits in practical use.

Biocompatibility refers to the ability of living tissues to interact with inert materials, typically concerning the compatibility between materials and the host [33]. Ensuring biocompatibility is crucial in developing novel bioactive materials to guarantee their safety and efficacy within biological systems. Biocompatibility involves not only the chemical stability and mechanical properties of materials but also their behavior in a biological environment, such as cytotoxic responses, immune reactions, and whether the degradation products of the materials are harmful to tissues [34]. Since elastomeric ligatures directly interface with the oral mucosa, we conducted oral mucosal irritation tests following the guidelines provided by the International Organization for Standardization (ISO) to assess their biocompatibility. Specifically, we first prepared an extract of the elastomeric ligatures, then soaked cotton swabs in the extract, and applied them to the oral cavity of test animals. After a certain period, samples were taken to analyze the surface morphology and histological changes in the buccal mucosa. The results in this study showed no significant differences in surface morphology and histological appearance between the buccal mucosa of the control group and the experimental group, indicating that the novel elastomeric ligatures possess good biocompatibility.

Monocytes are an essential component of the immune system, capable of differentiating into macrophages, which play a crucial role in immune defense [35]. Macrophages are not only responsible for protecting the body from injury or infection but also play a vital role in clearing necrotic tissue and cellular debris [36]. When exposed to external stimuli, macrophages initiate an inflammatory response by releasing a series of inflammatory mediators such as tumor necrosis factor-α (TNF-α), interleukin 6 (IL-6), and interleukin 1 beta (IL-1β) [37]. These inflammatory mediators recruit more immune cells to the site of inflammation, promoting immune cell infiltration and aiding in the resolution of inflammation and tissue repair processes. In this study, researchers extracted RNA from the buccal mucosa tissue of rats exposed to elastomeric ligature extracts and analyzed the expression levels of inflammatory markers *Tnfa*, *Il6,* and *Il1b* using quantitative PCR. By comparing the expression data of inflammatory markers between the DMAHDM group and the control group, the results showed no significant difference between the two groups. These indicated that the antimicrobial elastomeric ligature did not cause a significant inflammatory response in the oral mucosa of rats, demonstrating good biocompatibility.

It is worth noting that we included the elastomeric ligatures with 5% DMAHDM in our study design. In terms of antibacterial effect, 5% DMAHDM appeared to be the most effective, as its antibacterial properties were stronger than those of the elastomeric ligatures with 2% DMAHDM. However, as mentioned earlier, the purpose of the novel elastomeric ligatures we studied was to address the practical issue of enamel demineralization in orthodontic clinics, which necessitates meeting biosafety standards to fully protect patients. Since the cytotoxicity of the elastomeric ligatures increased significantly with the addition of 5% DMAHDM, despite its superior antibacterial effect, it lacks practical application value.

Indeed, there are some limitations in this study. The ultimate goal of developing these novel orthodontic elastomeric ligatures is their application in clinical orthodontic practice to address enamel demineralization in patients with fixed orthodontic appliances. While animal models can partially simulate the human oral environment, factors such as diet and species differences make the human oral environment more complex. Therefore, further clinical studies are essential to elucidate the actual preventive effects of these novel elastomeric ligatures on enamel demineralization.

For instance, the procedure could involve selecting a cohort of volunteers requiring orthodontic treatment and necessitating the extraction of 2–4 first premolars. At the initiation of the orthodontic treatment, specifically during the alignment and leveling phase prior to tooth extraction (at approximately 6 months), a split-study could be employed. Conventional elastomeric ligatures would be applied on one side, while novel elastomeric ligatures utilized on the contralateral side. Upon achieving dentition alignment and leveling, the premolars would be extracted and the degree of enamel demineralization analyzed to evaluate the practical preventive effect of the novel elastomeric ligatures on enamel demineralization.

## 4. Materials and Methods

This study was approved by the Animal Ethics and Welfare Committee of Beijing Stomatological Hospital Affiliated with Capital Medical University (no. KQYY-202403-006).

### 4.1. Incorporation of DMAHDM with Elastomeric Ligatures

The ultraviolet photochemical grafting technique was utilized for this process. Initially, elastomeric ligatures (Creative Dental, Ningbo, Zhejiang, CHN) were immersed in an acetone solution for 30 min to remove surface impurities, followed by 3–5 rinses with deionized water. The samples were then air-dried in a vacuum oven at room temperature. Next, a specific amount of benzophenone was added and completely dissolved in 20 mL of anhydrous ethanol. Various concentrations of DMAHDM (0, 0.5, 1, 2, and 5%) were added to the mixture, which was magnetically stirred for 20 min to prepare the grafting solution. The synthesis of DMAHDM followed the method described in a previous study [8].

The elastomeric ligatures were immersed in the grafting solution for 20 min, then removed, drained of excess liquid, and placed on an ultraviolet irradiation platform. They were exposed to ultraviolet light for 20 min, flipped, and irradiated for an additional 20 min. The grafted elastomeric ligatures were then rinsed with deionized water and subjected to ultrasonic cleaning in an ethanol solution for 10 min. Afterward, they were rinsed twice with deionized water and air-dried until a constant weight was achieved (Figure 1). Thus, novel elastomeric ligatures were prepared. The successful synthesis was validated using Fourier-transform infrared spectroscopy (FTIR, Thermo Fisher Scientific Nicolet iS20, Waltham, MA, USA) and water contact angle measurements (Dataphysics OCA20, Stuttgart, Germany). Parameters of FTIR: resolution: 4 cm^−1^; number of scans: 32; wavenumber range: 400–4000 cm^−1^. The method of water contact angle measurements: sessile drop, 3 μL; speed: 1 μL/s; range: 0–180°; accuracy: ±0.1°; 3 samples for each group.

### 4.2. Antibacterial Properties

Testing the antibacterial properties of individual elastomeric ligatures was unfeasible due to their limited volume. Therefore, a set of 20 elastomeric ligatures was selected for the evaluation of antibacterial properties.

#### 4.2.1. Biofilms Formation

*Streptococcus mutans* UA159 (Shanghai Bioresource Collection Center, Shanghai, China) were routinely cultured in brain–heart infusion broth (BHI; Difco, Sparks, MD, USA) with 0.2% sucrose at 37 °C under anaerobic conditions. After stable passaging, the elastomeric ligatures were co-cultured with the bacterial suspension to form biofilms on their surface.

#### 4.2.2. Lactic Acid Measurement and pH Measurement

The standard curve of lactic acid production was prepared using a Lactic Acid Assay Kit (Solarbio, Beijing, China). The elastomeric ligatures covered in biofilms at 24 h were rinsed with cysteine peptone water (CPW, Sigma-Aldrich, St. Louis, MO, USA) and then transferred to new 24-well plates. Buffered peptone water (BPW, Sigma-Aldrich, St. Louis, MO, USA) containing 0.2% sucrose was added to the biofilms to stimulate acid production for 3 h. Lactic acid production was monitored at OD570 nm using a microplate reader (SpectraMax Paradigm, San Jose, CA, USA). pH measurements were conducted using 5 mL of supernatant collected from each group at 8, 24, 48, and 72 h.

#### 4.2.3. Colony-Forming Units (CFUs) Counts

Each set of samples containing biofilms grown for 24, 48, and 72 h was transferred to a 2 mL test tube. The biofilms were detached using ultrasonic vibration and thoroughly mixed with a vortex shaker. Serial dilutions were then prepared in PBS and the viability of the microorganisms was assessed using BHI agar plates.

#### 4.2.4. Spectrophotometer Counting

After incubation periods of 24, 48, and 72 h, each group of bacterial suspensions was thoroughly mixed using a pipette. A 1 mL aliquot from each mixture was transferred into a cuvette. The spectrophotometer was calibrated to zero using BHI culture medium and the optical density of each sample was measured. This process allowed for the analysis of differences in bacterial quantities among the groups.

### 4.3. Cytotoxicity

#### 4.3.1. Preparation of Extract Solution

Following the guidelines established by the International Organization for Standardization (ISO), each group of elastomeric ligatures was immersed in a specified amount of Dulbecco’s Modified Eagle Medium (Gibco, Carlsbad, CA, USA) and incubated at 37 °C for 24 h to obtain the original extract solution.

#### 4.3.2. Cell Proliferation Activity

Mouse L929 fibroblasts (Procell Life Science&Technology, Wuhan, Hubei, China) were selected to analyze the effects of each group of extract solutions on cell proliferation activity. Third-generation fibroblasts were chosen for cultivation in different extract solutions. Cells were seeded at a density of 6000 cells per well in a 96-well plate and cultured for 24, 48, and 72 h. The proliferation activity was evaluated using the Cell Counting Kit-8 (Dojindo, Kumamoto, Japan). Additionally, fibroblasts cultured in the extract solution for 48 h were fixed with 4% paraformaldehyde solution and stained with SYTO9 dye (Invitrogen, Carlsbad, CA, USA) for fluorescence imaging to observe cell growth and morphological changes.

#### 4.3.3. Cell Migration

L929 cells were passaged and seeded into a 6-well plate at a density of 1 × 10^6^ cells/mL. After being cultured in serum-free medium for 24 h, a sterile 1 mL pipette tip was used to vertically scratch the cell monolayer. The suspended cells were gently washed away with PBS (phosphate-buffered saline, Gibco, Carlsbad, CA, USA) and the medium was replaced with different extract solutions for each group. Three parallel wells were set up for each group. Images of the scratch area were taken at the beginning and after 24 h to record the scratch. The effect of each extract solution on cell migration was analyzed to evaluate the cytotoxicity of each group of elastomeric ligatures.

### 4.4. Mechanical Testing of Elastomeric Ligatures with 2% DMAHDM

Firstly, an in vivo enamel demineralization model was established. Twelve female SD rats, weighing between 180 and 220 grams and aged 8 to 10 weeks, were selected. They were randomly divided into two groups, A and B (A: 4 rats; B: 8 rats). Following a 7-day adaptation period, the left buccal side of the upper first molars of rats in group B was bonded with two specially treated orthodontic buttons. Subsequently, novel elastomeric ligatures were fixed on the button of the left molar, while traditional elastomeric ligatures were fixed on the right molar. Rats in group A were provided with distilled water and a normal diet, while rats in group B were fed a high-sugar soft diet and water containing 1 mL of a bacterial suspension of *Streptococcus mutans* (5 × 10^8^ CFU/mL), which was renewed daily. Additionally, a daily application of 500 μL bacterial suspension was administered around the oral cavity of the rats using a cotton swab, ensuring coverage of the tongue and teeth. This procedure was carried out for 4 weeks to establish the in vivo enamel demineralization model.

The mechanical properties of elastomeric ligatures are particularly critical for orthodontic treatment. It is essential to ensure that imparting antibacterial properties to elastomeric ligatures does not compromise their mechanical properties. Therefore, the breaking strength and breaking length of the initial elastomeric ligatures in both the control group and the experimental group were tested. After four weeks of treatment in the in vivo enamel demineralization model, the rats were euthanized painlessly and the elastomeric ligatures from both sides were removed. These ligatures were then tested for tensile strength and elongation at break to evaluate their mechanical properties.

### 4.5. Mucosal Irritation

Based on the study mentioned above, a thorough screening process identified elastomeric ligatures with 2% DMAHDM exhibiting optimal antimicrobial properties and demonstrating no cytotoxic effects. Following the procedure outlined in Section 4.3.1, the extract solution was prepared. Twelve Sprague-Dawley (SD) rats were selected for the study. Cotton balls soaked in the extract solution, with a diameter of approximately 10 mm, were placed in one cheek pouch of the rats as the experimental group, while cotton balls soaked in 0.9% sodium chloride solution were placed in the opposite cheek pouch as the control group. After 2 h of contact, the rats were euthanized painlessly and representative tissue samples from the cheek pouches were fixed, embedded, sectioned, and stained with hematoxylin and eosin (HE). Histological differences between the experimental and control sides were then compared.

After thoroughly grinding another portion of the tissue (9 mm^2^), RNA extraction was performed following the instructions of the RNA extraction kit (Cowin Biotech Co.,Taizhou, Jiangsu, China). The extracted RNA was then reverse-transcribed into cDNA and subjected to real-time quantitative PCR (RT-qPCR) analysis. Semi-quantitative analysis was conducted on the inflammatory marker genes, tumor necrosis factor-α gene (*Tnfa*), interleukin 6 gene (*Il6*), and Interleukin 1 Beta gene (*Il1b*) in macrophages. The nucleotide sequences of related genes are shown in Table 4.

### 4.6. Enamel Demineralization

#### 4.6.1. Surface Microscopy and Energy Spectrum Analysis

For the rats euthanized as previously described, the maxillary bones were isolated and all soft tissues were meticulously removed. Prior to examination, the buttons were carefully detached from the teeth. Following that, the second molars, which were also contacted with elastomeric ligatures, underwent polishing before being coated with a 10 mA gold layer for subsequent analysis using scanning electron microscopy (SEM, ZEISS Gemini 300, Oberkochen, Germany) along with an energy dispersive spectrometer (EDS). The surface morphology of the demineralized enamel was scrutinized at an acceleration voltage of 3 kV. Additionally, a semi-quantitative analysis of the chemical element content of the enamel in various regions was conducted at an acceleration voltage of 15 kV and a current of 6.4 nA.

#### 4.6.2. Nanoindentation for Mechanical Properties

The second molars were embedded in a resin block, with particular attention given to ensuring the exposure of the labial enamel surface. The mechanical properties of the enamel surrounding the brackets were evaluated using a nanoindentation instrument (Bruker Hysitron TI980, Berlin, Germany). Employing a diamond Berkovich indenter, the maximum load was set at 25 mN, the loading time was 15 s, and the peak load holding time was 10 s. Subsequently, the elastic modulus (Er) and nanoindentation hardness (H) of the indentation point were determined.

### 4.7. Statistical Analysis

A one-way analysis of variance (ANOVA) was conducted to identify the significant effects of the variables, followed by the least significant difference (LSD) post hoc test at a significance level of 0.05. Statistical analyses were performed using SPSS software (version 26.0; IBM Corp., Armonk, NY, USA).

## 5. Conclusions

DMAHDM was combined with elastomeric ligatures by ultraviolet photochemical grafting and achieved significant antibacterial activity, reducing lactic acid production by 65.3% and total bacterial count by 50.0%. Elastomeric ligatures containing 2% DMAHDM exhibited excellent biocompatibility, causing no adverse effects on cells or mucosal tissues. In an in vivo animal model, the novel elastomeric ligatures significantly prevented enamel demineralization, improving elastic modulus and hardness by 73.2% and 204.8%, respectively. The novel elastomeric ligature with DMAHDM has great application prospects in preventing enamel demineralization during orthodontic treatment.

## Figures and Tables

**Table 1:** Antibacterial properties of the novel elastomeric ligatures.

Items	Mass Fraction of DMAHDM					*p* Value
	Blank	0.5%	1.0%	2.0%	5.0%	
Lactic acid production (mmol/L)	16.02 ± 0.60	12.20 ± 0.49	8.69 ± 0.87	5.56 ± 1.30	1.45 ± 0.63	<0.001
24 h OD600 value	0.70 ± 0.02	0.59 ± 0.02	0.50 ± 0.03	0.35 ± 0.03	0.16 ± 0.02	<0.001
48 h OD600 value	1.52 ± 0.05	1.42 ± 0.02	1.34 ± 0.02	1.07 ± 0.06	0.30 ± 0.03	<0.001
72 h OD600 value	1.72 ± 0.06	1.49 ± 0.03	1.40 ± 0.01	1.12 ± 0.04	0.31 ± 0.02	<0.001

Note. The results are presented as Mean ± SD.

**Table 2:** Cytotoxicity of the novel elastomeric ligatures.

Items (Each Group/Blank)/(%)	Mass Fraction of DMAHDM					*p* Value
	Blank	0.5%	1.0%	2.0%	5.0%	
24 h OD450 value	100.0 ± 3.0	128.1 ± 3.6	116.8 ± 8.2	92.6 ± 2.5	83.9 ± 9.1	<0.001
48 h OD450 value	100.0 ± 5.9	102.9 ± 8.1	111.0 ± 6.7	88.4 ± 6.1	51.1 ± 4.1	<0.001
72 h OD450 value	100.0 ± 9.3	110.8 ± 20.3	112.1 ± 20.9	77.5 ± 1.8	31.8 ± 6.1	<0.001

Note. The results are presented as Mean ± SD.

**Table 3:** Effects of the novel elastomeric ligatures on mechanical properties of rat molar enamel.

Items (Gpa)	Healthy	Control	DMAHDM	*p* Value
Elastic modulus (Er)	110.10 ± 6.46	43.65 ± 2.62	75.61 ± 4.57	<0.001
Hardness (H)	5.10 ± 0.54	0.62 ± 0.17	1.89 ± 0.65	<0.001

Note. The results are presented as Mean ± SD.

**Table 4:** Oligonucleotide primers applied in real-time polymerase chain reaction.

Primers	Nucleotide Sequence (5′–3′)
Gapdh	
Forward	5′-TCAGCAATGCCTCCTGCAC-3′
Reverse	5′-TCTGGGTGGCAGTGATGGC-3′
Tnfa	
Forward	5′-GGGCTCCCTCTCATCAGTTC-3′
Reverse	5′-CTTGGTGGTTTGCTACGACG-3′
Il6	
Forward	5′-ACCAAGACCATCCAACTCATC-3′
Reverse	5′-TTCTGACCACAGTGAGGAATG-3′
Il1b	
Forward	5′-TGCAGGCTTCGAGATGAAC-3′
Reverse	5′-GGGATTTTGTCGTTGCTTGTC-3′

## Data Availability

All data generated or analyzed during this study are available from the corresponding author upon reasonable request.
